# Efficacy and safety of YinQiSanHuang-antiviral decoction in chronic hepatitis B: study protocol for a randomized, placebo-controlled, double-blinded trial

**DOI:** 10.1186/s13063-020-04395-y

**Published:** 2020-06-05

**Authors:** Qing-Juan Wu, Wen-Liang Lv, Juan-Mei Li, Ting-Ting Zhang, Wen-hui Zhou, Qiang Zhang, Jiu-Chong Wang, Qing-Nan Wang, Ruo-Xuan Zhang, Xin Zhao, Si-Tong Chen, Shuang Liu, Gao-Hui Li, Zheng-Min Cao, Lei Xu, Jing Chen

**Affiliations:** 1 Guang’anmen Hospital of China Academy of Chinese Medical Sciences, Beijing, China; 2 Graduate School of Beijing University of Chinese Medicine, Beijing, China

**Keywords:** Chronic hepatitis B, Cirrhosis, Traditional Chinese medicine, Clinical trial, Efficacy

## Abstract

**Introduction:**

Chronic hepatitis B (CHB) is a global public health problem. Antiviral therapy is the primary treatment. Studies have shown that a combined therapy of traditional Chinese medicine (TCM) and conventional antiviral drugs has better efficacy than conventional antiviral for treatment of CHB. *YinQiSanHuang*-antiviral decoction (YQSH) is a TCM compound preparation that has shown an effect on anti-hepatitis B virus and on slowing progression of hepatitis B-related liver diseases. To evaluate the efficacy and safety of YQSH combined with entecavir and its preventive effect on hepatitis B cirrhosis, we designed this randomized, double-blind and placebo-controlled trial. The objective is that the combination of YinQiSanHuang-antiviral decoction with entecavir will reduce the annual incidence of liver fibrosis/cirrhosis to 1%.

**Methods:**

This is a multicenter, randomized, placebo-controlled, double-blinded trial involving five hospitals. A total of 802 patients are randomly allocated to two groups: the YQSH group (*n* = 401) or the placebo group (*n* = 401). The YQSH group receives YQSH with entecavir; the placebo group receives granules of placebo with entecavir. Patients receive treatment for 52 weeks and then are followed up for 52 ± 2 weeks. The primary outcome measure is the annual incidence of cirrhosis. The secondary outcome measures are hepatitis B virus DNA negative rate, hepatitis B surface antigen negative rate, hepatitis B e antigen seroconversion rate, liver function (alanine aminotransferase, aspartate aminotransferase , gamma-glutamyl transferase , alkaline phosphatase , serum albumin, and total bilirubin), spleen thickness, evaluation scores of patients’ clinical symptoms, and safety assessment. Outcomes will be assessed at baseline and after treatment.

**Discussion:**

Combination therapy could become a trend for treatment of CHB, and this trial expects to provide credible clinical evidence for the future combination of TCM and conventional antiviral drugs for the treatment of CHB.

**Trial registration:**

Chinese Clinical Trial Registry: ChiCTR1900021521. Registered on 25 February 2019.

## Background

Chronic hepatitis B (CHB) is a chronic viral infection caused by hepatitis B virus (HBV) and characterized by the persistence of hepatitis B surface antigen (HBsAg) for at least 6 months (with or without concurrent hepatitis B e antigen [HBeAg]). As a global health problem, more than 257 million people worldwide suffer from chronic HBV infection [1, 2]. In 2015, an estimated 887,000 deaths resulted from hepatitis B, mostly from cirrhosis and hepatocellular carcinoma (HCC, i.e., primary liver cancer) [3]; of these CHB is responsible for 30% of all deaths from cirrhosis and 40% of those from HCC [4–6]. China’s prevalence is one of the highest, with about 20 million CHB cases, accounting for 21.5% of the 93 million cases of HBV infection. Without timely testing and treatment, most CHB will develop into cirrhosis, HCC, or finally lead to death [7, 8]. It is estimated that in China there are 20–30 million people with CHB, 1 million with liver cirrhosis, and 0.3 million with HCC caused by hepatitis B [9]. Deaths due to HBV-related liver diseases in China (0.308 million deaths per year) account for more than 30% of the global mortality from HBV (0.887 million deaths per year) [10]. Antiviral therapy is the primary link to slow the conversion of CHB into cirrhosis and can effectively restore liver function and improve survival rate in patients with CHB [11, 12]. Currently, the US Food and Drug Administration (FDA) has approved two types of anti-HBV drugs: interferon (IFN) and nucleos(t)ide analogs (NAs, e.g., entecavir, lamivudine, telbivudine, adefovir, and tenofovir). Although NAs are well tolerated and exhibit an early and potent antiviral effect, the selection of resistant mutants and nephrotoxicity during long-term therapy limit their use [13, 14].

Chinese herbal medicine (CHM) is one of the most popular complementary and alternative therapies for CHB, and numerous studies have reported its anti-HBV effect. CHMs have two major characteristics: (1) they are flexible and adaptable in various kinds of herbal medicines, with complex compositions, which means they do not easily cause drug resistance; (2) they are taken from the natural environment, using rich, easily obtainable sources. CHMs are generally well tolerated for long-term treatment, which provides a good therapeutic effect in the prevention and treatment of CHB. A cohort study showed that the use of CHM is associated with significantly reversed cirrhosis and reduced HCC risk in patients with CHB [15]. Many studies have identified active ingredients in single herbs or in herbal formulas that have therapeutic effects on CHB, with associated anti-HBV, antifibrosis, liver protection, antitumor, antibacterial, antioxidant, anti-acute liver injury, and anti-HCC mechanisms [16–21]. An ideal anti-HBV drug should have good safety, good drug resistance, long-lasting effects, and no withdrawal rebound, and be able to stimulate host immune responses and effectively inhibit virus replication or even eliminate the virus [22, 23]. Studies have identified that CHM not only has antiviral effects, but it also can enhance the body’s immunity to improve its antiviral ability. For example, an extract of *Le-Cao-Shi*, which is a kind of TCM herb, can restrain the expression of duck hepatitis B model surface antigen (DHBsAg), hepatitis B e antigen (DHBeAg), and HBV DNA (DHBV DNA), correspondingly, in a HepG2.2.15 cellular model, and could also significantly inhibit the secretions of HBsAg and HBeAg [24]. Results from another study demonstrated that cordycepin, which is extracted from TCM, could work as an adjuvant to the hepatitis B vaccine, and this type of new vaccine simultaneously improves the humoral and cellular immunity of BALB/c mice without side effects [25]. *YinQiSanHuang*-antiviral decoction (YQSH) is a CHM formula that has been used for more than 30 years for CHB treatment. It has shown effects on anti-HBV DNA and increasing the level of alanine aminotransferase (ALT) in a small-scale clinical study [26]. Research results suggested that YQSH not only enhances the antiviral effect of entecavir, but also has a significant preventive effect on CHB-related cirrhosis. Thus, we designed this study and have set the objective as “the combination of YQSH with entecavir will reduce the annual incidence of liver fibrosis/cirrhosis to 1%” to test the effectiveness of this combination.

## Methods

### Study setting

**Fig. 1:**
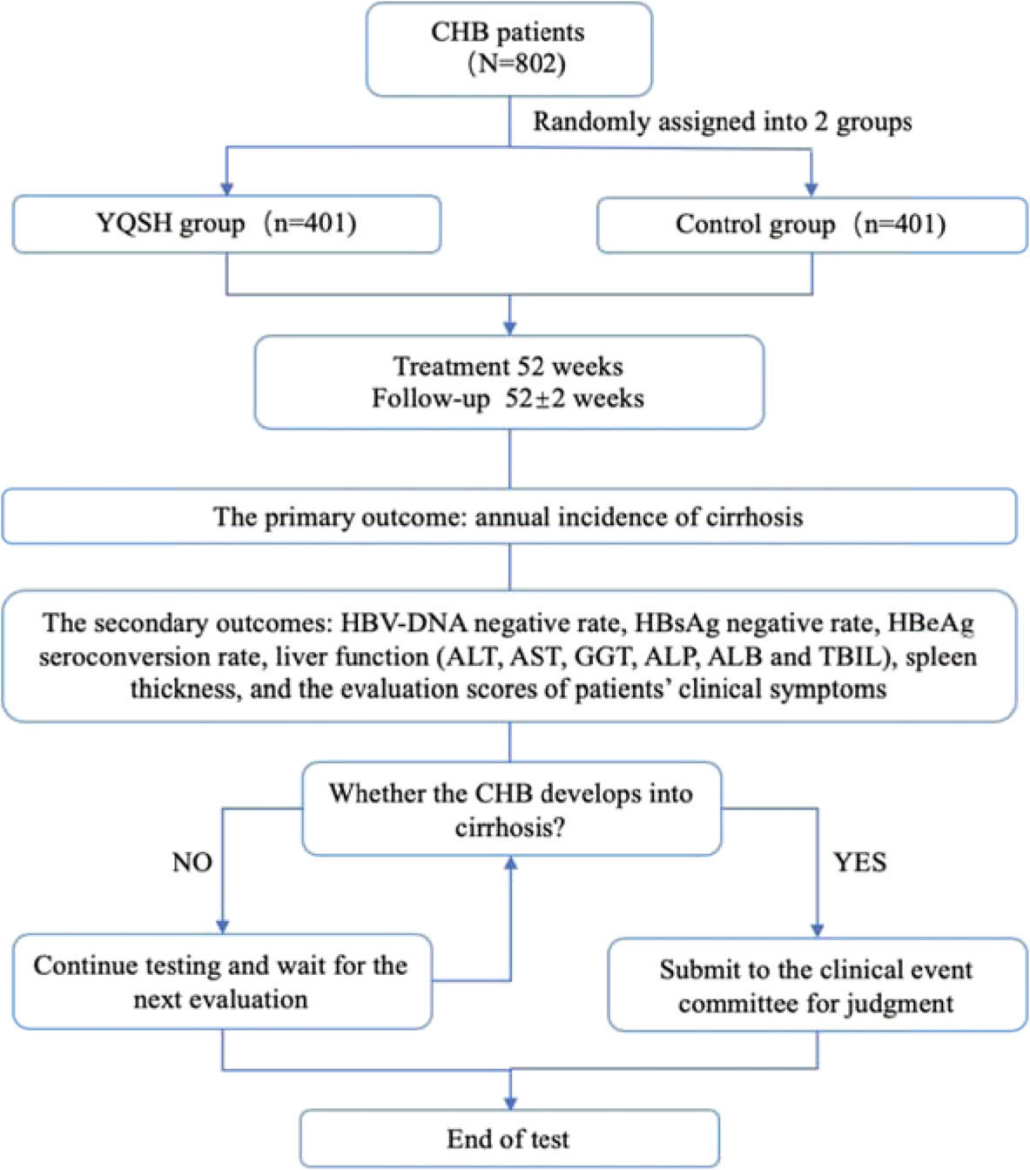
Flowchart of the randomized, placebo-controlled, double-blinded trial of YQSH for CHB. *CHB* chronic hepatitis B, *YQSH YinQiSanHuang*-antiviral decoction, *HBV* hepatitis B virus, *HBsAg* hepatitis B surface antigen, *HBeAg* hepatitis B e antigen, *ALT* alanine aminotransferase, *AST* aspartate aminotransferase, *GGT* gamma-glutamyl transferase, *ALP* alkaline phosphatase, *ALB* serum albumin, *TBIL* total bilirubin

A total of 802 patients will be recruited from five centers: the main responsible unit, Xixi Hospital of Hangzhou, will recruit 162 cases, Beijing ShunYi Traditional Chinese Medicine Hospital will recruit 160 cases, the Sixth People’s Hospital of Shenyang will recruit 160 cases, Beijing DiTan Hospital Capital Medical University will recruit 160 cases, Chengdu University of Chinese Medicine Affiliated Hospital will recruit 160 cases. Figure 1 shows the flowchart of the trial.

### Eligibility criteria

#### Inclusion criteria

The inclusion criteria are as follows: (1) patients who have a history of hepatitis B infection or HBsAg positive for more than 6 months, and their current HBsAg and/or HBV DNA levels are still positive; (2) patients who meet the criteria for antiviral indications in the “Asian-Pacific consensus statement on the management of CHB: a 2008 update” [27]; patients taking entecavir dispersible tablets may also be included. (This study will enroll patients who have taken entecavir previously; conversely, patients who have taken immune stimulatory drugs or other antiviral drugs within 3 months will not be enrolled.); (3) patients between 18 to 65 years old; (4) patients showing syndromes of liver stagnation and spleen deficiency and dampness in TCM. For the TCM diagnostic criteria, we refer to “National Standards for TCM Clinical Diagnosis and Treatment in the People’s Republic of China” [28] and the “Medical Consensus of diagnosis and treatment of cirrhosis with integrated TCM and Western medicine” [29], published by the Digestive System Diseases Committee, Society of Integrated Traditional Chinese and Western Medicine.

#### Exclusion criteria

The exclusion criteria are as follows: (1) patients with cirrhosis; (2) patients with liver cancer; (3) patients in acute and chronic hepatitis with non-HBV infection, autoimmune hepatitis, primary biliary cirrhosis, primary sclerosis cholangitis, inherited metabolic liver disease, drug or toxic hepatitis, or alcoholic liver disease; (4) pregnant or lactating women or women planning to become pregnant during the study period; (5) patients who are allergic to the test drugs; (6) patients who have mental disorders and cannot cooperate with the study, or patients with epilepsy in unstable status; (7) patients with severe systemic disease related to the heart, brain, lung, kidney, or hematopoiesis; (8) patients with alcoholism or other unsuitable conditions for enrollment. Patients who are already using TCM will not be enrolled unless they have stopped using TCM for more than 3 months.

### Interventions

#### Description

All patients will have a screening test during the screening period in clinic if they meet the inclusion criteria. The screening evaluation includes the patient’s general status, disease-related symptoms and signs, and corresponding laboratory tests, including the following: urine pregnancy test (women of childbearing age), HBV DNA, liver function, FibroScan, liver B-mode ultrasound or magnetic resonance imaging (MRI)/computed tomography (CT), and other examinations. The test group (YQSH group) will receive YQSH formula granules 5 g (first brewed with 150–200 ml water) twice a day, combined with entecavir 0.5 mg once a day. The control group (placebo group) will receive YQSH placebo formula granules 5 g (first brewed with 150–200 ml water) twice a day, combined with entecavir 0.5 mg once a day. The entecavir was produced by ChiaTai TianQing Pharmaceuticals in Jiangsu, China (production batch number H20100019). The main components of YQSH, totaling 14 kinds of herbs, are shown in Table 1. The test drugs are made into Chinese medicine formula granules. Before taking YQSH, the mixed formula granules are boiled with 150–200 ml water for 3–5 min. The YQSH placebo is made of excipients, thinners, coloring agents, flavoring agents, and fried malt, and is similar to YQSH in shape, color, smell, and taste.

**Table 1:** Main components of *YinQiSanHuang*-antiviral decoction

Chinese name	Latin name	English name	Pharmacological action	Main active ingredient	Original producing area	Medicinal part	Dosage (g)
*Huang Qi*	*Astragalus propinquus* Schischkin	Radix Astragali	Promotes liver cell growth, anti-liver fibrosis, antiviral, regulates immunity	Astragaloside (I,V, III), calycosin	Neimenggu, China	Rhizome	12
*Yin Chen*	*Artemisia capillaris* Thunb.	Virgate wormwood herb, capillary wormwood herb	Lowers blood lipids to treat fatty liver, reduces alcoholic liver damage, inhibits the replication of hepatitis B virus DNA	Capillin, capillene, capillanol, capillarisin, 6, 7-dimethylsculetin	Shanxi, China	Aboveground part of the plant	12
*Huang Qin*	*Scutellaria baicalensis* Georgi	Baical skullcap root	Anti-hepatocyte inflammation, anti-hepatocyte apoptosis, anti-hepatocyte mitochondrial lipid peroxidation, regulates immunity	Baicalein, neobaicalein, skullcap flavone II, baicalin, wogonin	Hebei, China	Rhizome	3
*Huang Lian*	*Coptis chinensis* Franch.	Coptis root	Anti-hepatocyte mitochondrial lipid peroxidation, inhibits hepatoma cell proliferation, prevents liver fibrosis	Berberine, coptisine, epiberberine, berberrubine, palmatine	Sichuan, China	Tuber root	3
*Huang Bai*	*Platycladus orientalis* (Linn.) Franco	Bark of Chinese corktree	Inhibits immune response, selectively inhibits HBAg, anti-inflammatory	Berberine, phellodendrine, magnoflorine, jatrorrhizine, palmatine	Sichuan, China	Dry bark	3
*E Zhu*	*Curcuma aeruginosa* Roxb. [*C. zedoaria* Rosc.]	Rhizome curcumae	Inhibits hepatoma cell proliferation, induces apoptosis of liver cancer cells, anti-liver fibrosis	Volatile oil (turmerone, borneol, curcumol), curcumene, curdione, turmeric	Guangxi, China	Tuber root	6
*Bie Jia*	*Trionyx sinensis* (Wiegmann)	Turtle shell	Anti-liver fibrosis, promotes immunity, anti-hepatocyte injury	Collagen, *Trionyx sinesis* polysaccharides, amino acids (aspartic acid, threonine, glutamic acid), calcium carbonate, calcium phosphate	Hubei, China	Carapace	3
*Jiao Shan Zha*	*Crataegus pinnatifida* Bunge var. major N.E. Br.	Hawthorn fruit	Lowers cholesterol, anti-bacterial, anti-hypertensive	Epicatechin, quercetin, hyperoside, chlorogenic acid, anthocyanin, ursolic acid	Shandong, China	Fruit	15
*Bai Shao*	*Paeonia lactiflora* Pall.	Radix paeoniae alba	Anti-hepatocyte injury, anti-liver fibrosis, anti-fatty liver	Paeoniflorin, oxy-paeoniflorin, benzoylpaeoniflorin, albiflorin, paeoniflorigenone	Anhui, China	Rhizome	12
*Ling Xiao Hua*	*Campsis grandiflora* (Thunb.) K. Schum.	Trumpet creeper flower	Anti-oxidation, inhibits thrombosis, anti-inflammatory	Apigenin, β-sitosterol	Jiangsu, China	Flower	6
*Bai Zhu*	A*tractylodes macrocephala* Koidz.	Largehead atractylodes rhizome	Inhibits liver cancer cell metastasis, promotes cellular immune function, inhibits the activating of metabolic enzymes	Volatile oil (humulene, β-elemol, α-curcumene, α-atractylone, 3β-acetoxyatractylone), sesquiterpene lactone compounds (atractylenolide, 8β-ethoxyatractylenolide-II), polyacetylene(14-acetyl-12-senecioyl-2E,8Z,10E-atracetylentriol)	Zhejiang, China	Tuber root	9
*Fu Ling*	*Poria cocos* (Schw.) Wolf.	Tuckahoe	Enhances cellular and humoral immunity, inhibits the DNA synthesis of tumor cells, inhibits hepatocyte necrosis, anti-tumor	Pachymic acid, tumulosic acid, pachymic acid methyl ester, pachy-man, pachymaran	Yunnan, China	Dry sclerotia	9
*Chai Hu*	*Bupleurum chinense* DC.	Red thorowax root	Anti-liver fibrosis, inhibits acute liver injury, inhibits proliferation of liver cancer cells, promotes apoptosis of liver cancer cells, anti-liver injury	Volatile oil (pentanoic acid, hexanoic acid, heptanoic acid, 2-heptenoic acid)	Hebei, China	Rhizome	6
*Bai Hua She She Cao*	*Hedyotis diffusa* Willd.	Spreading hedyotis herb	Enhances hepatocyte immunogenicity, anti-tumor, inhibits proliferation of liver cancer cells, promotes apoptosis of liver cancer cells	Asperuloside, asperulosidic acid, deacetylasperulosidicacid, geniposidic acid, scandoside	Guangxi, China	Whole plant	12

#### Modifications

If a patient shows abnormal liver function (such as ALT > 2*ULN) during the trial, this should prompt temporary or permanent cessation of treatment, and the investigator can add hepatoprotective drugs such as silibinin and/or other drugs according to the patient’s condition. At the same time, the researchers will record and follow up the situation of the patient closely. If the patient’s ALT returns to normal after liver protection treatment, with normal values for direct bilirubin and international normalized ratio (INR) at the same time, then he/she may have a chance to continue the trial; otherwise, the patient will stop the trial permanently for safety reasons. If the following conditions occur, subjects should discontinue the trial: (1) poor compliance, irregularly taking medicine, failure to revisit or revisit on time; (2) some combined diseases or complications, or deterioration during the trial; (3) subject self-withdrawal; (4) combining trial drug with other drugs, or not taking test drugs according to research regulations; (5) lost contact; (6) cannot provide complete information.

We also list the following specific stopping criteria. (1) Serious safety problems occur during the test; then the test should be stopped in a timely manner. (2) The drug is found to have no clinical value during the trial; then the trial should be stopped to avoid delaying the effective treatment of subjects. (3) It is found during the trial that there is a major error in the clinical trial protocol and it is difficult to evaluate the effect of the drug; or there is a well-designed protocol with important deviations in the implementation, and it is difficult to continue to evaluate the efficacy and safety of the drug. (4) The sponsor requests to stop the trial (for funding reasons, management reasons, etc.). (5) The State Food and Drug Administration of China orders the trial to be stopped for some reason. (6) The test is suspended due to *force majeure* reasons.

#### Adherence

The study will be regularly monitored by a Clinical Research Associate (CRA) in accordance with the corresponding standard operating procedure. The CRA will help ensure that the research center adheres to the research protocol, arranges the supply of research drugs, and ensures that the drugs are kept under appropriate conditions in accordance with instructions. At the beginning of the trial, the researcher would emphasize the importance of compliance to the subjects, and require the subjects to bring back the drug packages (regardless of the remaining drugs) when they visit the research center. At the same time, we will establish an online platform to make immediate contact with the patients, and contact them at least twice a month to learn the patients’ situations and remind them to actively return to the clinic. For those patients who participated effectively during the treatment but could not complete the entire course, and those lost to follow-up, these data will be included in the efficacy statistics and should be analyzed intentionally.

#### Concomitant care

Other antiviral medicines, such as tenofovir or lamivudine, must not be taken during the trial. If non-antiviral medicines are combined, they will be recorded in the case report form (CRF). If the subjects need other treatment or concomitant care, they should contact the doctor in advance.

### Outcomes

#### Primary outcome

The primary outcome is the annual incidence of cirrhosis (the examination methods include instantaneous liver elastic hardness test, abdominal B-mode ultrasound test, or abdominal MRI/CT imaging).

The primary outcome is evaluated before the treatment, at 52 weeks of treatment, and at the 52 ± 2 weeks follow-up visit.

#### Secondary outcomes

The secondary outcomes include HBV DNA negative rate, HBsAg negative rate, HBeAg seroconversion rate, liver function (ALT, aspartate aminotransferase [AST], gamma-glutamyl transferase [GGT], alkaline phosphatase [ALP], serum albumin [ALB], and total bilirubin [TBIL]), spleen thickness, and the evaluation scores of patients’ clinical symptoms.

These indicators are observed before the treatment, at 26 weeks of treatment, at 52 weeks of treatment, at the 26 ± 2 weeks follow-up period, and at the 52 ± 2 weeks follow-up period.

#### Safety outcomes

The safety outcomes include adverse events (AEs), laboratory tests (liver function, kidney function, routine blood tests, routine urine tests, etc.), electrocardiography (ECG), basic vital signs, and physical examination.

**Fig. 2:**
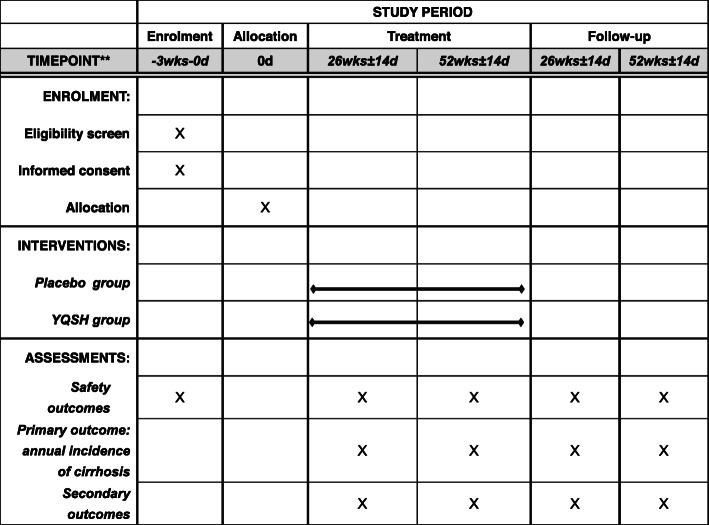
Standard Protocol Items: Recommendations for Interventional Trials (SPIRIT) schedule of enrollment, interventions, and assessments

The basic vital signs are body temperature, blood pressure, respiration, and heart rate; laboratory tests include renal function tests, blood urea nitrogen (BUN), creatinine (Cr), and routine blood, stool, and urine tests. These biological indicators are monitored starting from the group allocation of the patients until the end of follow-up (Fig. 2).

#### Participant timeline

The treatment period is 52 weeks, and the follow-up assessment period will last for 52 ± 2 weeks. We include two figures to show the participant timeline more clearly. (See Figs. 1 and 2.)

#### Sample size

The aim of this study is to reduce the annual incidence of cirrhosis from 2% ~ 10% [30] to 1% in patients with CHB. Therefore, according to the sample size estimation formula for comparison of two sample rates, the incidence of target events is less than 0.2 (or 0.3) or greater than 0.8 (or 0.7), shown as follows:

$$ \mathrm{n}=\frac{{\left({u}_{\alpha }+{u}_{\beta}\right)}^2}{2{\left({\mathit{\sin}}^{\hbox{-} 1}\sqrt{p_e}{\hbox{-} \mathit{\sin}}^{\hbox{-} 1}\sqrt{p_c}\right)}^2} $$

Here *p*_*e*_ and *p*_*c*_ represent the incidence rates of the test group (YQSH group) and placebo group (control group), respectively. The positive event rate (*p*_*c*_) in the control group is 5%, while the target event rate (*p*_*e*_) in the test group is set to 1%. Since the values of p_e_ and p_c_ are small, the degree is measured in radians, α = 0.05, and β = 0.10. In this study, a two-sided test was chosen, *u*_0.05_ = 1.96, *u*_0.10_ = 1.282, *p*_*e*_ = 0.05, *p*_*c*_ = 0.01. The calculated sample size of each group is approximately 334 cases. Therefore, allowing for 20% attrition, the total number of patients required for this trial is 334*(1 + 20%)*2 = 802 cases, with 401 in each group.

#### Recruitment

Outpatients in clinics are the main recruitment targets. Posters and online publicity containing a brief introduction to the trial and the contact information of the researchers will also be used for recruitment. Before enrollment, each patient is provided with a complete and comprehensive description of the test procedure, its purpose, potential AEs, and expected benefits. Subjects are also informed that they may withdraw from the trial. If a patient agrees to participate in the trial, he/she will sign two informed consent forms, one kept by the patient and the other kept by the researcher. Subjects with involuntary or incomplete autonomy can also enter the trial after the consent of the ethics committee, and the informed consent form will be signed by their guardian.

### Allocation

#### Sequence generation and implementation

A central randomization system (CRS) is applied to ensure a completely randomized design. The randomized system mainly includes the following modules: subject screening, randomization, emergency unblinding, drug formulation, drug supply management, and other functional modules. The central randomization principle is as follows: The researcher uses the screening module to enter some basic information for the subject (such as date of birth, gender) and obtain the subject’s unique identification number (SIN). Firstly, the researcher confirms that the patient meets the inclusion criteria, logs into the CRS, inputs the general information for the subject, generates the random number, and fills in the eCRF. Secondly, drug distributors apply for the drug number from the CRS according to the random number. Finally, the drug senders verify the code on the drug package with the number in the system, and then the drugs are given to the patients.

#### Concealment mechanism

In this study, the CRS is used to centrally control the allocation of the entire randomization scheme. The “central randomization” method is used to conceal the allocation: When researchers determine that the subjects are qualified, the researchers log into the CRS, enter some basic information for the subjects, and obtain the subjects’ SIN. Then, the CRS will assign the subjects’ random numbers and drug numbers according to a designed blind table. In order to make the blind method effective and reduce drug loss, the random numbers are separated from the drug numbers in this system. Although the random number and the drug number are different, their corresponding treatment plans are consistent within the system.

#### Blinding and emergency unblinding

This is a double-blind trial. The blinding method is set up and implemented by the Medical Statistics Center of Tianjin University of Traditional Chinese Medicine. Neither the study researchers nor the subjects know the medication grouping. In the course of the trial, a scientific and strict management implementation system and feasible operation methods are used. All the subjects are put under a standardized observation with their clinical symptoms carefully recorded. Adverse reactions are carefully observed, and emergency unblinding is required for serious adverse reactions.

A regular supervision, inspection, and return system is used to ensure the implementation of the double-blinding method.

Unblinding will be performed at the end of the trial to perform a statistical analysis of all the data. The outcome assessment will be blinded. After all the research data have been entered and locked, the third party participants who saved the blinding codes and the researchers will jointly unblind and submit the database to the statistical analyst. When the entire statistical analysis is completed, statistical analysis and clinical trial summary reports will be written by the researchers.

#### Data collection plan and data management

The investigator will prepare original documents for each subject who was randomized into the study, and the information will be recorded in the CRF. All research results (personal data, test documents, etc.) that appear in the original medical records will be completely confidential within the scope allowed by law. Subject names will not appear in the CRF; only the name initials and the random number will be shown. The content should be comprehensive and accurate so as to record all examination results and other relevant data. The research center shall keep these documents properly for 5 years after the end of the research. The researcher will authorize the relevant regulatory agency to directly access all research-related documents.

There are principles for handling loss to follow-up: (1) if the loss is due to adverse reactions, the data will be recorded in the adverse reaction statistics; (2) if the loss is due to ineffectiveness, the data will be included in the efficacy statistics; (3) for those patients who were effective during the treatment but could not complete the entire course, and those lost to follow-up, these data will be included in the efficacy statistics and should be analyzed intentionally.

### Statistics

#### Outcomes

For the statistical analysis comparing the primary outcome incidence between the two groups, we will use the χ^2^ test and set *P* < 0.05 (95% confidence interval) as statistically significant. For the secondary outcomes, the measurement data are expressed as mean ± standard deviation, the count data are expressed as frequency and percentage (*f*, %), and the frequency or percentage of the efficacy evaluation index is converted into frequency and percentage (*f*, %). For comparison of the mean between the two groups, the homogeneity test is performed first. If the variances are equal, the *t* test is used. If the variances are unequal, the non-parametric *t* test is used. The measurement data of each group before and after treatment are compared using the paired *t* and/or *t* test. The comparison of grid table count data will be performed using the χ^2^ test, and the comparison of rank data will use the rank sum test. *P* < 0.05 will be used as the statistical difference. The data analysis will be performed using SPSS 19.0 statistical software.

The baseline is defined as the last observation data before the first medication, which includes demographic characteristics and clinical baseline data: age, sex, vital signs (height, weight, temperature, heart rate, blood pressure, breathing), clinical symptom scores, HBV DNA, liver stiffness (FibroScan/Fibrotouch), entecavir treatment history, and CHB-related diseases.

#### Additional analyses

There are currently no plans to do subgroup analyses or sensitivity analyses.

#### Analysis population and missing data

We will use three methods for statistical analysis of the data:

- Full analysis set (FAS). According to the intention-to-treat (ITT) principle, all randomized subjects’ data will enter the FAS. For subjects who withdraw from the study early for various reasons, the missing data will be imputed using the method of last observation carried forward (LOCF).
- Per protocol set (PPS). The study’s PPS will consist of the indicator data for those who enter the study and complete treatment and follow-up, whose medication compliance is 80–120%, who took no combined medication that affected the effectiveness evaluation during the study period, and who have complete evaluation index data and no major test protocol violations.
- Safety set (SS). The SS includes those subjects who received at least one treatment after randomization.

### Monitoring

#### Formal committee and confidentiality

A data monitoring committee (DMC) will monitor the trial in accordance with the corresponding standard operating procedure, and is independent from the sponsor. The DMC will be allowed to evaluate the quality and integrity of the study. Before the start of a clinical trial, uniform training should be conducted for all the participants, including training in Good Clinical Practice (GCP), research protocols, the electronic data capture (EDC) system, central stochastic systems, and the use of scales. The DMC will assess the capabilities of the research centers and collect information about institutional facilities and technical equipment. During the period of study, the DMC is responsible for verifying the clinical research records with the original records and for resolving any problems that arise during the trial. The DMC will monitor whether written consent and dated informed consent forms have been obtained from all subjects. The DMC will also monitor that the research center adheres to the research protocol, arranges the supply of research drugs, and ensures that the drugs are kept under appropriate conditions in accordance with instructions. Each center should submit the main indicators to the clinical endpoint committee to be evaluated by uniform standards. The principal investigator and authorized researcher should review, electronically sign, and date the eCRF.

#### Interim analysis

The DMC has access to the interim results and makes the final decision with the sponsor and researcher on whether to terminate the trial.

### Harms

Any adverse medical events that occur during treatment and follow-up, regardless of whether or not there is a causal relationship with the test medicines, should be considered as an adverse event (AE) and recorded in the specified AE table of the case report form (CRF). When filling out the AE report forms, it is necessary to record in detail the occurrence, time, severity, duration, measures taken, and outcomes of the AE. If serious AEs occur during the trial, emergency treatments should be taken immediately and the event reported to the lead researcher of the trial, the ethics committees, and the China State Food and Drug Administration Safety Supervision Department within 24 h. All AEs should be tracked until the adverse symptoms disappear or the researchers confirm that further follow-up is no longer needed.

If the subject has an injury that is directly related to this study during the course of treatment, and it is confirmed by medical identification, the research team will pay the subject’s medical expenses. For serious AEs caused by drug-related injuries, the research team will give the subject certain compensation in accordance with relevant national laws and regulations, and the compensation costs will be borne by Guang’anmen Hospital.

### Auditing

Beginning with enrollment, each research center will receive auditing visits every 3 months. After the data are recorded, a professional medical review will be conducted to compare the data entered in the CRF with the original data, in order to ensure the quality of the data, the clinical logic, and general medical terms for the description. The researcher will properly keep the data to protect the rights and privacy of subjects, the documents of the clinical trial shall be preserved and managed in accordance with the requirements of the GCP, and the database will be maintained by an EDC system. The auditing procedures are performed independent of the investigators.

## Discussion

Hepatitis B is a hidden killer. In the course of viral infection for decades, the virus carrier (patient) may show only mild symptoms, which are difficult to detected, but the infection may ultimately develop into liver cancer [31]. Currently, direct-acting antiviral agents (DAAs) including NAs and IFN can control HBV, but they cannot eliminate it completely. Therefore, pre-treatment is the best way to prevent the deterioration of CHB and cirrhosis. In the theory of TCM, there is a viewpoint of “preventive treatment of disease”, which means precautions should be taken in advance, when disease has not occurred or has just occurred, to guard against further disease development or deterioration. In China, CHM has been used for more than 2000 years and has played an important role in the prevention and treatment of disease. A TCM decoction is generally composed of various Chinese herbs in a certain proportion, and is characterized by a multi-target effect. YQSH is a TCM decoction based on the theory of preventive treatment of disease, and in early small-scale clinical observation it has suggested a potential effect on delaying the development of CHB-related cirrhosis.

At present, long-term HBV DNA inhibition or HBsAg negative status is considered to be the best surrogate endpoint for antiviral therapy in patients with CHB or cirrhosis associated with HBV [23, 32–34]. Studies have reported that combined therapy is superior to conventional antiviral therapies [35–37], in that it can not only enhance antiviral ability, but it can also reduce the accompanying symptoms, improve the quality of life, and prolong the life of patients [38, 39]. Thus, combination therapy could become a trend in CHB treatment. Looking back on the clinical research on combination therapy in CHB in recent years, there have been mostly single-center, single-field, and small-sample studies, which makes their conclusions less credible. Therefore, this trial protocol was designed into a multicenter, randomized, double-blind, placebo-controlled clinical trial, with follow-up as long as 52 ± 2 weeks, to verify the clinical efficacy and safety of the combination therapy of YQSH + entecavir, with the expectation that it will provide credible clinical evidence for the future combination of TCM and Western medicines for the treatment of CHB.

There are also some limitations to the study. Due to restrictions in research project funds and trial periods, the follow-up period could not be longer, and thus additional randomized controlled trials with long-term follow-ups are warranted. For combination therapy, there are still some problems to be clarified, such as how the drugs are combined, when is the best time for combination therapy, when to stop the drug, whether therapy can be repeated after stopping the drug, and how to cope with recurrence after drug withdrawal. It is assumed that more combination therapies will be explored in the future based on different mechanisms.

### Trial status

The protocol version number is 1, and it was finalized in October 2018. Recruitment began on 21 October 2019. The approximate date when recruitment will be completed is December 2021. If we should amend the protocol, we will communicate with the investigators, ethics committee, trial registries, and other relevant parties.

## Abbreviations

ALB: Serum albumin
ALP: Alkaline phosphatase
ALT: Alanine aminotransferase
AST: Aspartate aminotransferase
BUN: Blood urea nitrogen
CHB: Chronic hepatitis B
CHM: Chinese herbal medicine
CRF: Case report form
CRS: Central randomization system
DAA: Direct-acting antiviral agent
ECG: Electrocardiography
FDA: Food and Drug Administration
GCP: Good Clinical Practice
GGT: Gamma-glutamyl transferase
HBV: Hepatitis B virus
HCC: Hepatocellular carcinoma
IFN: Interferon
NA: Nucleos(t)ide analog
TBIL: Total bilirubin
TCM: Traditional Chinese medicine
YQSH: YinQiSanHuang-antiviral decoction
